# Patient with PSEN1 Glu318Gly and Other Possible Disease Risk Mutations, Diagnosed with Early Onset Alzheimer’s Disease

**DOI:** 10.3390/ijms242015461

**Published:** 2023-10-23

**Authors:** YoungSoon Yang, Eva Bagyinszky, Seong Soo A. An

**Affiliations:** 1 Department of Neurology, Soonchunhyang University College of Medicine, Cheonan Hospital, Cheonan 31151, Republic of Korea; astro76@naver.com; 2 Department of Industrial and Environmental Engineering, Graduate School of Environment, Gachon University, Seongnam-si 13120, Republic of Korea; 3 Department of Bionano Technology, Gachon Medical Research Institute, College of Bionano Technology, Gachon University, Seongnam-si 13120, Republic of Korea

**Keywords:** PSEN1, ABCA7, SORL1, TOMM40, GRN, risk factor, early onset Alzheimer’s disease, gene interactions

## Abstract

In this manuscript, we introduced a French EOAD patient in Korea who carried the presenilin-1 (*PSEN1*) Glu318Gly mutations with four possible risk variants, including sortilin-related receptor 1 (*SORL1*) Glu270Lys, ATP-binding cassette subfamily A member 7 (*ABCA7*) Val1946Met, translocase of outer mitochondrial membrane 40 (*TOMM40*) Arg239Trp, and granulin (*GRN*) Ala505Gly. The patient started to present memory decline and behavioral dysfunction in his early 60s. His brain imaging presented amyloid deposits by positron emission tomography (PET-CT). The multimer detection system (MDS) screening test for plasma for amyloid oligomers was also positive, which supported the AD diagnosis. It was verified that *PSEN1* Glu318Gly itself may not impact amyloid production. However, additional variants were found in other AD and non-AD risk genes, as follows: *SORL1* Glu270Lys was suggested as a risk mutation for AD and could increase amyloid peptide production and impair endosome functions. *ABCA7* Val1946Met was a novel variant that was predicted to be damaging. The *GRN* Ala505Gly was a variant with uncertain significance; however, it may reduce the granulin levels in the plasma of dementia patients. Pathway analysis revealed that *PSEN1* Glu318Gly may work as a risk factor along with the *SORL1* and *ABCA7* variants since pathway analysis revealed that *PSEN1* could directly interact with them through amyloid-related and lipid metabolism pathways. *TOMM40* and *PSEN1* could have common mechanisms through mitochondrial dysfunction. It may be possible that *PSEN1* Glu318Gly and *GRN* Ala505Gly would impact disease by impairing immune-related pathways, including microglia and astrocyte development, or NFkB-related pathways. Taken together, the five risk factors may contribute to disease-related pathways, including amyloid and lipid metabolism, or impair immune mechanisms.

## 1. Introduction

Early-onset Alzheimer’s disease (EOAD) is a relatively rare form of Alzheimer’s disease (AD) before 65 years of age. Three main genes were identified as causative factors for EOAD: amyloid precursor protein (*APP*, NC_000021.9), presenilin 1 (*PSEN1*, NC_000014.9), and presenilin 2 (*PSEN2*, NC_000001.11). Among them, the majority of EOAD-related variants were reported in the *PSEN1* gene with more than 300 mutations (http://www.alzforum.org/mutations/psen-1 (accessed on 20 April 2023)). Variants in *PSEN1* present diverse clinical phenotypes. The typical EOAD hallmarks were intracellular amyloid deposits and extracellular neurofibrillary tangles with fast disease progression. Atypical disease phenotypes were also reported, including motor impairments (spastic paraparesis, Parkinsonism, and ataxia), language dysfunctions, or personality changes [1,2]. Besides the classical EOAD-related genes, additional AD risk factor genes were also suggested to impact early-onset AD onset through amyloid-related mechanisms, including the sortilin-related receptor *(SORL1*) [3] or ATP-binding cassette (ABC) transporter A7 (*ABCA7*) [4] genes. *SORL1* could play a role in APP trafficking from the cell membrane to the Golgi complex. Normally, *SORL1* would protect against amyloid processing by APP transport. *SORL1* may also reduce the amyloid load by directing the amyloid peptides to the lysosomes [5,6]. The *ABCA7* gene plays an important role in the lipid-related pathways by protecting against amyloid aggregation through inducing phagocytosis and enhancing amyloid clearance [7]. Mutations in *ABCA7* and *SORL1* could be involved in the loss-of-function mechanisms, leading to elevated amyloid beta aggregations [4,8]. Translocase of outer mitochondrial membrane 40 (*TOMM40*) is a mitochondrial channel-forming protein involved in protein transport into the mitochondria. Coding and non-coding mutations (such as polyT repeat in intron 6) would be risk factors for AD. *TOMM40* variants were reported to be related to AD through mitochondrial dysfunction or inflammation-related mechanisms [9,10]. Mitochondria plays a significant role in maintaining synaptic and neuronal functions. Furthermore, mitochondria could be essential in cellular respiration and regulating the bioenergetics of neurons. Impaired mitochondrial pathways could lead to neurological dysfunction and AD through multiple pathways, such as abnormal calcium homeostasis, the production of reactive oxygen species, and mitophagy [11,12]. One of the associations between non-AD-related genes and AD was through granulin (*GRN*), a causative gene for frontotemporal dementia (FTD). *GRN* played a significant role in immune-modulation neurodevelopment and synapse formation, and the reduced expression of *GRN* in the brain was suggested as a risk factor for AD [13,14]. Inflammation may also play a significant role in neurodegeneration and AD progression. Microglia and macrophage activation were observed during the aging process and in several neurodegenerative diseases. In AD, microglia may be activated by amyloid deposits. Amyloid overload may result in abnormal inflammasome formation and neurodegeneration. Inflammatory markers (pro- and anti-inflammatory cytokines/chemokines) were observed in the brain and biological fluids of patients with cognitive decline and AD [15].

In this study, we described a 65-year-old EOAD patient who carried a known *PSEN1* mutation, Glu318Gly. This mutation may not be a causative factor for EOAD, but it may act as a potential risk factor. Additional rare variants in other AD-related genes from the proband patient included *SORL1* (Glu270Lys), *ABCA7* (Val1946Met), and the *TOMM40* gene (Arg239Trp), with a rare variant in *GRN* (Ala505Gly). We suggested that *PSEN1* Glu318Gly may impact the neurodegenerative pathways by interacting with the above-mentioned possible risk genes. This study was approved by the Institutional Review Board of Soonchunhyang University College of Medicine, Cheonan Hospital (IRB number: 2023-02-038).

## 2. Case Presentation

The patient was a 68-year-old male patient of French nationality (from the 5th arrondissement of Paris) who presented memory dysfunctions and behavioral problems in his early 60s. Initially, he returned home without any treatment. A year before the genetic analysis, his cognitive decline worsened, and he visited the hospital with the symptoms of an inability to do his daily activities (including cleaning himself), talk to himself while looking in the mirror, and say words that did not fit the situation and were gibberish. The neurological examination was performed, and no abnormality was observed in kinesthetic symptoms. Misidentification syndrome was observed in his behavior at the front of the mirror. The family history of the patient remained unclear, but he revealed that his mother passed away with dementia at the age of 73. The father of the patient passed away at the age of 65, without any disease phenotype. The patient did not have any siblings. Other family members denied the genetic test or any other health information.

From the Mini-Mental State Examination, his score was 1 with a Global Deterioration Scale of 6 and a CDR (Clinical Dementia Rating) of 3, indicating a status of severe dementia. The proband patient’s cognitive function deteriorated quickly 5 years after the first symptoms. The symptoms worsened faster compared to general Alzheimer’s dementia. The patient’s *APOE* genotype was E3/E4.

**Figure 1:**
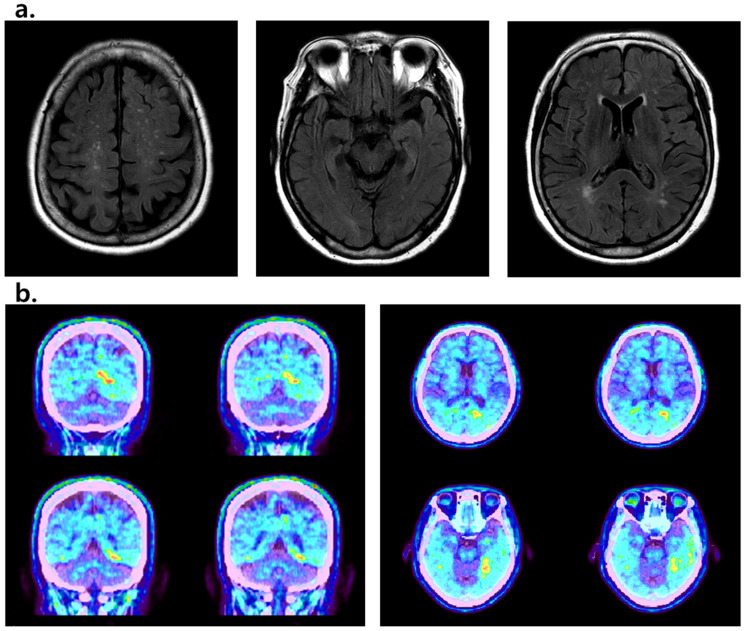
(**a**) MRI data of the proband patient, which showed diffuse cortical atrophy and enlarged ventricles; (**b**) PET-CT data from the patient showed amyloid deposits in different brain areas.

The diffuse cortical atrophy and moderate ventricle enlargement were observed from the brain magnetic resonance imaging (MRI) examination. Next, the positron emission tomography (PET)-CT (Amyloid-Florapronol Brain) revealed the abnormal amyloid deposits in the gray matter of both parietal and temporal lobes (Figure 1). The multimer detection system-oligomerized amyloid beta (MDS-OAβ, PeoplBio Inc., Seongnam, Republic of Korea) assay presented a score of 0.99, which was higher than the cut-off of normal persons (0.90). Results from analyses of MRI, PET-CT, and MDS-OAβ suggested the diagnosis of Alzheimer’s disease. He was prescribed drug treatments for Alzheimer’s disease. For cognitive dysfunction, donepezil and memantine were used in combination, and there was no significant change even after administration. However, the patient’s abnormal behavior, including misidentification syndrome, significantly improved after administration of quetiapine. Although the patient was admitted to the hospital at the age of 68, he was already in a state of severe dementia at the time of admission, and the family member described that the symptoms started 5 years ago, which meant that he would be a patient with EOAD when he was 63 years old.

## 3. Methods

The sample of the patient was received as whole blood, and the total DNA was purified from white blood cells using a Quiagen blood kit (Seoul, Republic of Korea). The whole exome sequencing (WES) and its analysis were performed with the patient’s genomic DNA on the Illumina platform by Macrogen (https://www.macrogen.com (accessed on 1 March 2023), Seoul, Republic of Korea). The sequencing data were visualized by Integrative Genomics Viewer (IGV) software 2.52 [16]. Other possible genes were selected for analysis, which could impact different neurodegenerative diseases, including risk factors for AD, Parkinson’s disease, frontal temporal disease, amyotrophic lateral sclerosis, and vascular diseases (Supplementary Table S1). Mutation-carrying genes found in the patient were screened by pathway analysis tools, including STRING and Cytoscape Cluego software, version 3.10.0 (https://apps.cytoscape.org/apps/cluego, accessed on 20 April 2023) [17]. The 3D structures of *PSEN1*, *SORL1*, *ABCA7*, *TOMM40*, and *GRN* were modeled by the Phyre2 tool (http://www.sbg.bio.ic.ac.uk/phyre2/html/page.cgi?id=index (accessed on 20 April 2023)). The possible structural alterations resulting from the rare variants were modeled by the Discovery Studio 3.5 Visualizer tool.

## 4. Results

The patient did not carry any pathogenic mutations in the *APP*, *PSEN1*, or *PSEN2* genes. However, several potential risk factor variants were observed in the patients by whole-exome analysis. A *PSEN1* variant, Glu318Gly (g. 73673178A>G; c.953A>G), was found in the patient. Additional rare variants were discovered in AD risk genes, such as *ABCA7* Val1946Met (g. 1063667G>A, c.5836G>A), *SORL1* Glu270Lys (g. 121367627G>A, c.808G>A), and *TOMM40* Arg239Trp (g. 44900801 C>T). Furthermore, a rare variant Ala505Gly (g. 42429809C>G; c.1514C>G) was observed in the *GRN* gene. All variants were heterozygous (Table 1, Supplementary Figure S1). Details on the variants found in the patient were summarized in Supplementary Table S2.

*PSEN1* Glu318Gly seemed like a relatively rare variant in East Asians since its GnomAD frequency was 0.00005017. In other populations, it may be more common; for example, its frequency was 0.006148 in South Asians, 0.006045 in Latinos, 0.01927 in non-Finnish Europeans, 0.03394 in Finnish Europeans, and 0.002927 in African populations. The in silico prediction by SIFT and PolyPhen2 suggested that *PSEN1* Glu318Gly was a benign variant and not conserved among vertebrates. However, CADD scoring predicted the variant to be damaging, with a score of 22,3. The *SORL1* Glu270Lys and the novel *ABCA7* Val1946Met variants were predicted to be pathogenic with all of these tools. *TOMM40* Arg239Trp was suggested as a possibly damaging variant by PolyPhen2, but the SIFT scores were low for this variant. The CADD score was high for *TOMM40* Arg239Trp, with a score of 25.1. However, *GRN* Ala505Gly received was predicted to be benign by PolyPhen2, and SIFT and CADD scores were also relatively low (15.92), suggesting that it may not be a strong pathogenic factor for AD.

**Figure 2:**
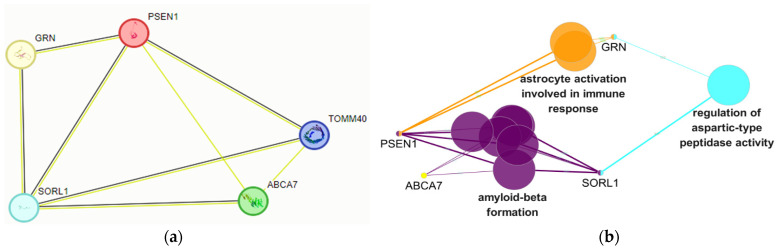
(**a**) STRING pathway analysis of patients: the significant pathogenic effects of the variants in all the above genes could be suggested from the direct interconnectivity among the above genes. The black edges mean that co-expression may occur in the case of the above genes. The green edges mean that these genes may interact based on “text mining”, and have possible associations based on literature [18]. (**b**) ClueGo interactions of the patient: variants in the above genes would affect amyloid-beta formations, astrocyte activations, and aspartic-type peptidase activities. Edge thickness indicates the score of the interaction. Different colors show different pathways. The colors of the edges and nodes and the length of nodes are customizable [19].

Pathway analysis with STRING and ClueGo predicted that PSEN1 could interact with the other rare variant carrier genes. In STRING analysis, direct interaction was found between *PSEN1* and *GRN*, *SORL1*, *ABCA7*, and *TOMM40* genes. (Figure 2a).

However, Cluego interactions failed to find any interaction with *PSEN1* and *TOMM40*, neither directly nor indirectly. (Figure 2b). *PSEN1* may interact directly with *ABCA7* and *SORL1* through amyloid-related mechanisms, including amyloid beta formation. A direct interaction was found between *PSEN1* and *GRN* through immune-related mechanisms, such as astrocyte activation, which were involved in the immune response. Interestingly, *GRN* and *SORL1* would also interact by regulating aspartic-type peptidase activity. (Direct interaction between *GRN* and *SORL1* was also observed by STRING networking). Aspartic peptidases, or aspartyl proteases, contain two catalytic aspartates and may be involved in protein degradation. Both the alpha-beta and gamma secretases could have aspartyl protease activity [20,21].

**Figure 3:**
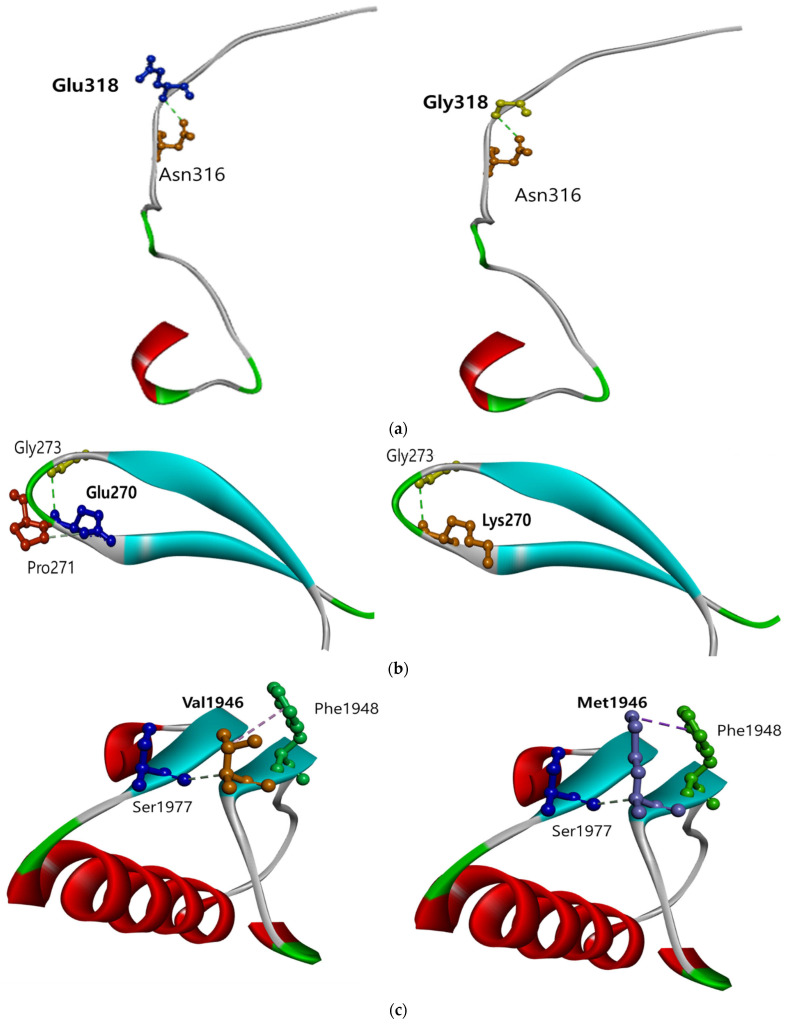

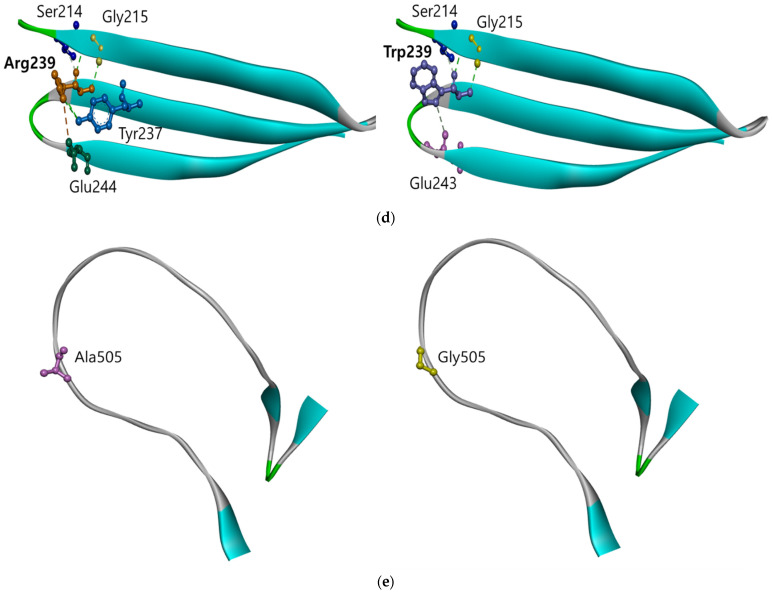
Structure predictions for rare variants. (**a**) PSEN1 Glu318Gly (**b**) SORL1 Glu270Lys (**c**) ABCA7 Val1946Met (**d**) TOMM40 Arg239Trp (**e**) GRN Ala505Gly. Blue means beta sheet structure, while red means alpha helix structure. Green means it is a kink inside helix/loop. The grey part is the loop area of protein. The colors of the highlighted amino acids are customizable.

Structure predictions were performed on all rare variants to probe the altered intramolecular interactions. Even though the prediction of *PSEN1* Glu318Gly structure (Figure 3a) revealed limited significant disturbances, both Glu318 and Gly318 may form a hydrogen bond with Asn318 in the large non-conservative loop of PSEN1. However, it may be possible that the small, hydrophobic, and uncharged glycine may result in abnormal flexible loop motion.

*SORL1* Glu270Lys (Figure 3b) was located in a small loop connecting two beta sheets. The normal Glu270 formed hydrogen bonds with Pro271 and Gly273, and Lys270 disconnected the hydrogen bond with Pro271, which may result in stress between the two beta sheets. Furthermore, the glutamic acid had a negatively charged side chain, while the lysine had a positively charged side chain, which may result in abnormal electrostatic interactions between SORL1 and its putative interacting partner.

*ABCA7* Val1946Met (Figure 3c) was located in a beta sheet region. Both Val1946 and Met1946 formed hydrogen bonds with Ser1977 and hydrophobic interactions with Phe1948. The larger amount of methionine could result in dysfunction inside the ABCA7 protein by forming an altered hydrophobic interaction.

*TOMM40* Arg239Trp (Figure 3d) was located inside the pore-forming region of the TOMM40 protein. The normal Arg239 could form hydrogen bonds with Ser214, Gly215, Tyr237, and Glu244. In the case of Trp239, the contact with Ser214 and Gly215 may remain, but the hydrogen bonds with Tyr237 and Gln244 were lost with the newly formed hydrogen bond with Gly243. The indol side chain of tryptophan may result in intramolecular stress through abnormal protein interactions and impaired beta sheet motion inside the TOMM40 protein.

The *GRN* Ala505Gly (Figure 3e) was located on a loop region of granulin protein. Neither Ala505 nor Gly505 were predicted to interact with any other residues nearby.

## 5. Discussion

We presented an EOAD patient who carried the *PSEN1* Glu318Gly mutation with additional potential risk factor variants for AD or other diseases, including *SORL1* Glu270Lys, *ABCA7* Val1946Met, *TOMM40* Arg239Trp, and *GRN* Ala505Gly. *PSEN1* Glu318Gly was located in the C-terminal region of the long hydrophilic loop (HL-6) of the PSEN1 protein. In this region, several other probable non-pathogenic mutations were observed, including Lys311Arg, Pro355Ser, and Arg377Trp [22]. Several studies were performed on *PSEN1* Glu318Gly to determine its role in neurodegeneration (Table 2), suggesting that *PSEN1* Glu318Gly may not be a causative mutation for EOAD. From multiple AD genetic studies of both EOAD and LOAD patients and controls, segregation was not found in several cases with low penetrance. Additionally, Glu318 was not a conserved residue in PSEN1 among vertebrates [23,24,25]. Taddei et al. (2002) screened Glu318Gly in Australian EOAD and LOAD patients in comparison with controls and suggested a higher frequency in EOAD cases (8.7%) in comparison to LOAD patients or controls (3.1 and 2.2, respectively). In addition, a significant difference was found in mutation frequency in the comparison of familial AD cases and age-matched controls [26]. A study by Albani et al. (2007) also observed a similar association between FAD cases and the Glu318Gly mutation [27]. It may be possible that the *PSEN1* Glu318Gly variant would increase the risk of LOAD only in participants carrying the *APOE* E4 allele [28,29,30]. However, other studies failed to find an association between the *APOE* E4 allele and *PSEN1* Glu318Gly, suggesting that the two risk factors may contribute independently to AD progression [25]. One investigated study of the role between Glu318Gly and DLB suggested a possible contribution to the onset of dementia with Lewy Body (DLB) in 10 cases between 40 and 85 years of age. The family history of most DLB patients was clinically negative. AD pathology may also be present in these patients [31]. Additional studies screened the association between *PSEN1* Glu318Gly and other potential risk factors, *SORL1* and *ABCA7*. Coppola et al. (2021) presented two EOAD cases with Glu318Gly. One of the patients, who developed EOAD (presented memory and attention disorder) at the age of 55, carried a *SORL1* Thr833Ile, and the other patient had *ABCA7* Asp679Tyr variants. They were both predicted as probable damaging variants by PolyPhen2 and SIFT tools. This study suggested that the *SORL1* and *ABCA7* variants may impact amyloid-related processes along with *PSEN1* Glu318Gly [32]. Further studies reported that *PSEN1* Glu318Gly co-existed with other *PSEN1* variants, such as Leu291Pro [33] or Lys311Arg [34].

Taken together, *PSEN1* Glu318Gly may not be responsible alone for the onset of EOAD. However, it may interact with other potential risk factor variants. The proband patient carried additional rare variants in *SORL1*, *ABCA7*, *TOMM40*, and *GRN* genes, which may contribute to disease progression [32,33,34]. The *SORL1* Glu270Lys was a rare variant in SORL1, located in the VPS10 domain of the SORL1 protein, which would affect the abnormal APP sorting. *SORL1* Glu270Lys was discovered in American, European, and Saudi Arabian patients [44,45]. Initially, conflicting reports were available about whether the mutation could impact AD. Fernandez et al. (2016) suggested that it may be a possible risk factor for EOAD [46]. However, other association studies, including those by Verheijen et al., 2016 [45], Sassi et al. (2016) [47], and Campion et al. (2019) [48], and Holstege et al. (2023) [49], failed to find associations with Glu270Lys and AD. Segregation of *SORL1* Glu270Lys with disease was not proven definitively. In one Spanish AD family, mutation did not segregate with disease since it was missing in one of the AD patients [50]. However, in a Caribbean family, segregation was observed between *SORL1* Glu270Lys and AD [46]. Cell studies of *SORL1* Glu270Lys with HEK293 mutant cell lines suggested that this mutation may impact AD onset by observing the increased levels of Ab42 and Ab40 and soluble APP-beta. Furthermore, even though the expression of *SORL1* was not changed at the surface of the mutant cells, the co-immunoprecipitation studies revealed the reduced binding ability of SORL1 to APP [46,51]. Expressing Glu270Lys in iPSC also revealed abnormal APP processing, resulting in impairment in endosomal trafficking and enlarged early endosomes in mutation-carrier neurons [52].

The proband patient also carried a novel variant in *ABCA7* V1948M, which was predicted to be damaging by PolyPhen2, SIFT, and CADD tools. Missense variants of *ABCA7* were suggested to increase the risk for both early and late-onset AD. *ABCA7* plays an important role in amyloid and lipid metabolisms, including cholesterol homeostasis. The variants could also impact immune functions such as phagocytosis and amyloid clearance. The Val1946Met mutation was located in the C-terminal region, close to the Walker B domain of the ABCA7 protein, which was a highly conserved area in ABCA7 for ATP binding and hydrolysis. Val1948Met could disturb ATP regulations by generating extra stress in the Walker B motif and contribute to neurodegeneration by resulting in impairment in the above metabolisms [7,53,54]. ABC-transporter proteins were suggested to impact ATP binding and hydrolysis, as well as lipid and cholesterol homeostasis and transport inside the brain [55]. Studies on ATP-binding cassette protein A1 (ABCA1 or ABCA7) suggested that mutations in the nucleotide binding domains (including Walker A and B motifs) of ABC transporter proteins could result in reduced ATP binding and abnormal interaction between ABCA proteins and apolipoproteins, such as (apolipoprotein A-I), leading to abnormal cholesterol transport [56]. Since beta- and gamma-secretases are located in the cholesterol rafts of transmembrane domains, high cholesterol levels may increase the APP cleavage into amyloid peptides. Furthermore, high cholesterol levels could inhibit alpha-secretase activity [55,57].

*TOMM40* was verified as a possible risk factor for AD with 5′ upstream of the *APOE* gene. *TOMM40* could play a significant role in mitochondrial transport by forming a mitochondrial channel protein. Mitochondrial degeneration was suggested to play a role in AD onset as an early hallmark of the disease prior to the appearance of neurofibrillary tangles [9,10]. Expression of *TOMM40* may be changed in different areas of the brain of AD patients in comparison to controls, where up-regulations were observed in the frontal lobe of AD patients [10]. Besides the non-coding variants [58], missense mutations (such as F113L and F131L) in *TOMM40* may also impact the AD risk. It was suggested that these variants may be involved in AD through inflammation-related mechanisms. Taiwanese carriers of these variants were associated with increased pro-inflammatory cytokines (including IL-6, IL18) in their plasma. Interestingly, increased microglia activations and inflammasome formations were found in patients with *TOMM40* F113L or F131L [9]. Even though *TOMM40* Arg239Trp was a rare variant in a conserved region of *TOMM40*, its association with AD or any forms of neurodegeneration remained unclear [59].

Furthermore, we found a rare *GRN* variant, Ala505Gly. Previously, this mutation was found in Italian patients [60,61]. One of the cases had motor neuron disease at the age of 62 with predominant subcortical multi-infarct vascular encephalopathy from MRI analysis. Family history may not be ruled out since the patient’s mother had late-onset dementia. Ala505Gly may alter an ESE site in the *GRN* exon. Plasma GRN was lower than normal, 76.2 ng/mL, but not low enough for the diagnosis of frontotemporal dementia (normal range > 100 ng/mL, the mean value in FTD patients was 61.2 ng/mL) [60,61]. This mutation was predicted to be benign by PolyPhen2 and SIFT, and CADD scores were also below 20, suggesting that the mutation may not be a damaging variant. Pathway analysis revealed common mechanisms between *PSEN1* and *GRN* through immune-related mechanisms. Granulin has been verified as an immunomodulator protein, which could impact both pro-inflammatory and anti-inflammatory mechanisms. For example, progranulin degradation into granulin fragments may induce pro-inflammatory IL8 production. In addition, by interacting with tumor necrosis factor receptor 1 (TNFR1), the GRN could impact the production of anti-inflammatory cytokines (IL10) through the ERK1/2 and PI3K/AKT pathways. These processes could also play a significant role in inhibiting pro-inflammatory pathways, including TNF-alpha production and the NF-κB inflammatory process [62,63]. Furthermore, granulin mutations may result in abnormal astrocyte and microglia-related mechanisms, such as their development and activation [64,65]. PSEN1 could also impact inflammatory pathways. PSEN1 dysfunction may be associated with abnormal inflammatory processes, such as the induction of pro-inflammatory factors and impaired glial functions [66]. PSEN1 was also verified as a regulator of the NF-κB pathway, since PSEN1 overexpression was associated with increased pro-inflammatory mechanisms. The immunoregulation by PSEN1 may be independent of gamma-secretase-related pathways [67]. Mutant PSEN1 may impact astrocyte functions, leading to reduced amyloid clearance [68,69].

According to reference databases (such as GnomAD), these variants are relatively rare or even absent (*ABCA7* Val1946Met) in general populations. It may occur rarely that all of these variants exist in one patient. Unfortunately, we were unable to perform segregation analysis on these five variants since all living family members refused the genetic test. In the future, we are planning to perform cell studies to analyze the potential effects of these mutations on cell lines.

Lastly, *PSEN1* Glu318Gly may not result in a disease phenotype by itself, but it may be a risk for disease, especially late-onset AD. However, the patient carried four additional missense mutations (*SORL1* Glu270Lys, *ABCA7* Val1948Met, *TOMM40* Arg239Trp, and *GRN* Ala505Gly), which could play a role in the disease pathogenicity. SORL1 Glu270Lys was already verified to be a risk factor for AD. Next, the novel Val1946Met mutation in *ABCA7* may impact the disease phenotype. Pathway analysis revealed that both *PSEN1*, *SORL1*, and *ABCA7* may be closely related and share several common pathways, including lipid metabolism and amyloid-related pathways. The common disease-related pathways of *PSEN1* Glu318Gly and *GRN* Ala505Gly may not be ruled out either through astrocyte or microglia-related pathways, leading to reduced amyloid clearance. *PSEN1* and *TOMM40* could possibly interact and play a role in AD onset through mitochondria-related mechanisms. Although it is difficult for a gene to predict the progression of the disease, our patient’s cognitive function progressed faster and worsened rapidly in just 5 years, revealing a different pattern than general Alzheimer’s dementia. Furthermore, the results of the MDS-OAb of Abeta oligomer test revealed that the patient had higher levels of Abeta oligomers in the blood, which is typical in the majority of AD patients [70,71], suggesting funneling effects on the Abeta pathway from the reporting mutations.

## Figures and Tables

**Table 1:** Summary of potential risk variants in the proband patient. NA means not available.

Variant	rsID	1000Genomes	Conserved?	GnomAD	SIFT	PolyPhen2	CADD
*PSEN1* Glu318Gly	rs17125721	0.00559105	No	0.01813	0.135, B	0.03, B	22.3
*ABCA7* Val1946Met	NA	NA	yes	NA	0.031, D	0.805, D	24.7
*SORL1* Glu270Lys	rs117260922	0.00778754	yes	0.01507113	0.008, D	0.998, D	31
*TOMM40* Arg239Trp	rs142412517	0.000199681	yes	0.000058	0.14, B	0.648, D	25.1
*GRN* Ala505Gly	rs780159686	NA	yes	0.0000731	0.257, B	0.444, B	15.92

(D means damaging, B means benign).

**Table 2:** Studies on *PSEN1* Glu318Gly mutation and potential disease risk. NA means, not available.

Study	Disease	Result	Biomarker Data	Suggestion	Population
Taddei et al. 2002 [26]	EOAD	More common in EOAD than in controls	NA	Glu318Gly may be similar risk factor like APOE E4	Australia
Albani et al. 2007 [27]	EOAD	Significant association between variant and familial EOAD	Fibroblasts: reduced Ab42/Ab40 ratio	Glu318Gly may be risk factor for EOAD, but further studies were needed	Italy
Mattila et al. 1998 [23]	Familial and sporadic AD	Observed in both controls and patients	NA	Not causative factor	Finnish
Dermaut et al. 1999 [24]	AD and other dementias	Variant was common in Dutch population	NA	May not be causative or risk factor	Netherlands
Aldulo et al. 1998 [35]	AD, LOAD and vascular dementia	Most common in EOAD, but also appeared in controls	NA	May not be causative or risk factor, has low penetrance	Spain
Zekanowski et al. 2004 [36]	AD, PD	No significant differences between disease and controls	NA	May not be causative or risk factor, has low penetrance	Poland
Helisalmini et al. 2000 [37]	Familial and sporadic AD	Detected in both patients and controls	NA	Possible risk factor in Finnish population	Finland
Perrone et al. 2020 [25]	EOAD and LOAD	Detected in patients and controls	CSF Ab1-43 was reduced, sAPPα and sAPPβ reduced	Found mild association with AD, independently from APOE genotype	Belgium
Mathioudakis et al. 2023 [38]	AD, MCI	Appeared in patients and controls	NA	May not be causative or risk factor, has low penetrance	Greece
Hippen et al. 2016 [39]	AD	APOE E4 carriers with Glu318Gly had higher risk for AD, but not significantly	NA	This study did not provide strong support on association of Glu318Gly and AD	USA
Jin et al. 2012 [40]	AD and control	Appeared in patients and controls	NA	May not be causative or risk factor, has low penetrance	Spain
Benitez et al. 2013 [29]	AD	More frequent in AD patients than in controls Glu318Gly variant with Aβ deposition was observed in APOE E4 carriers.	High CSF-Tau and P-Tau	Glu318Gly with APOE E4 allele could be associated with more senile plaques and faster cognitive decline	USA
Nho et al. 2016 [28]	AD	Increased risk for AD io E4 carriers	lower CSF Aβ1-42 and higher CSF tau	LOAD risk factor in E4 carriers	USA
Sala Frigerio 2015 [41]	AD	Appeared in patients and controls	NA	May not be causative or risk factor, has low penetrance	Belgium
Abdala 2014 [30]	AD	Association was found between AD and Glu318Gly	NA	No association between APOE E4 and Glu318Gly Glu318Gly variant may increase AD risk	Brazil
Lee et al. 2014 [42]	AD	Associated with dementia in case of cases vs controls	NA	Possible risk factor	Caribbean Hispanic
Day et al. 2019 [43]	AD	Age of onset was earlier expected	NA	Possible risk modifier?	USA
Geiger et al. 2016 [31]	DLB	Frequency was higher than in controls	NA	Possible association with DLB?	USA
Coppola et al. 2021 [32]	AD	Found in two EOAD patients	NA	May interact with other risk factors in SORL1, ABCA7	Italy
Eryilmaz et al. 2021 [33]	AD	Co-existed with a pathogenic L291P mutation in PSEN1	NA	May impact the disease course in the presence of pathogenic mutation	Turkey
Bisceglia et al. 2022 [34]	MCI	Co-existed with PSEN1 Lys311Arg	CSF Tau and amyloid levels were normal	Glu318Gly and Lys311Arg may result in risk to neurodegeneration	Italy

## Data Availability

Not applicable.

## Supplementary Materials

The supporting information can be downloaded at: https://www.mdpi.com/article/10.3390/ijms242015461/s1.
