# Supplementary material for: Patient with PSEN1 Glu318Gly and Other Possible Disease Risk Mutations, Diagnosed with Early Onset Alzheimer’s Disease
Source: Int J Mol Sci. 2023 Oct 23;24(20):15461. doi: 10.3390/ijms242015461 (PMC10607718; doi:10.3390/ijms242015461)
Supplement: Supplementary file 1 [file ijms-24-15461-s001.zip › ijms-2642793-supplementary.pdf]

Supplement figure

Supplement figure S1. Sequencing data of the five variants, found the patient

PSEN1 Glu318Gly

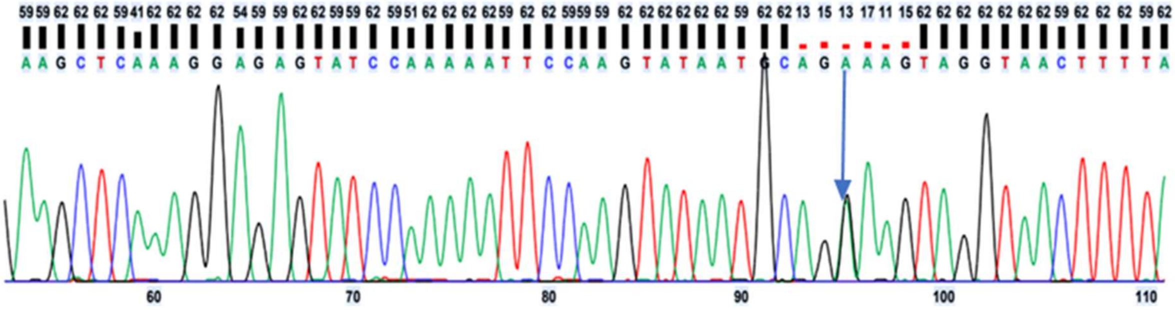

GRN Ala505Gly

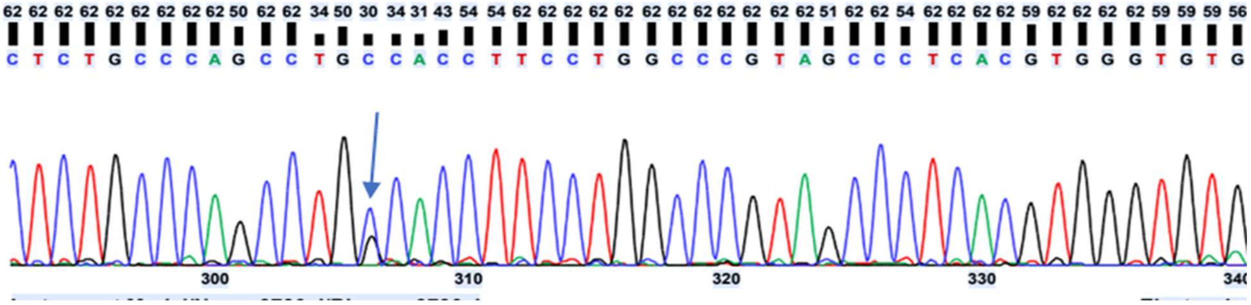

ABCA7 Val1946Met

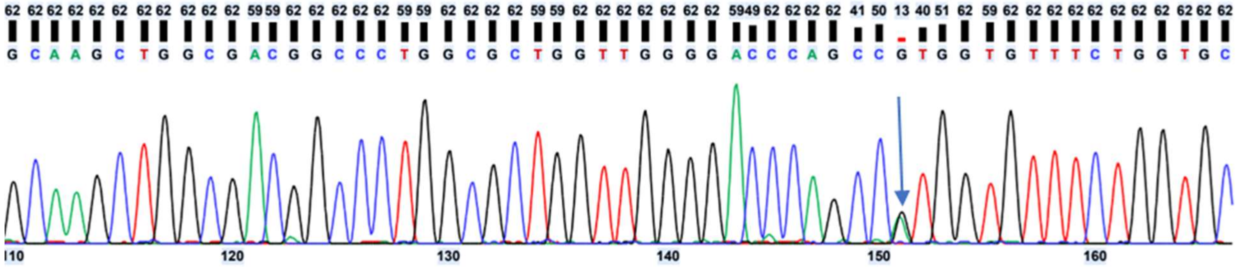

SORL1 Glu270Lys (3’ sequencing). The patient also carried a silent variant H269H

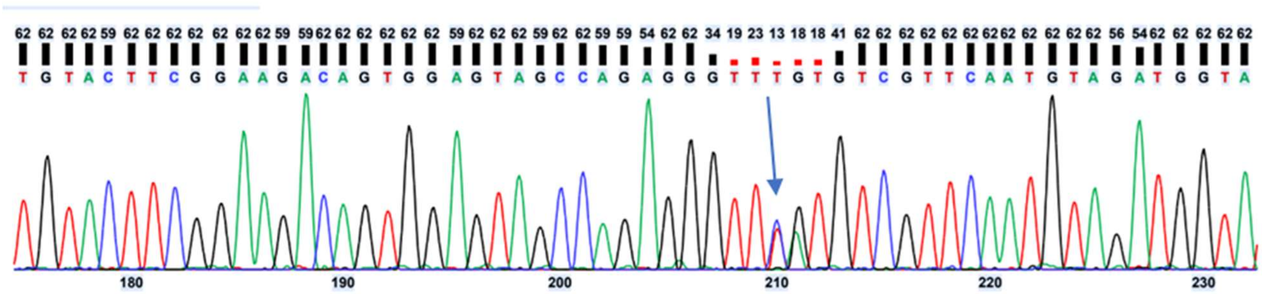

TOMM40 Arg239Trp

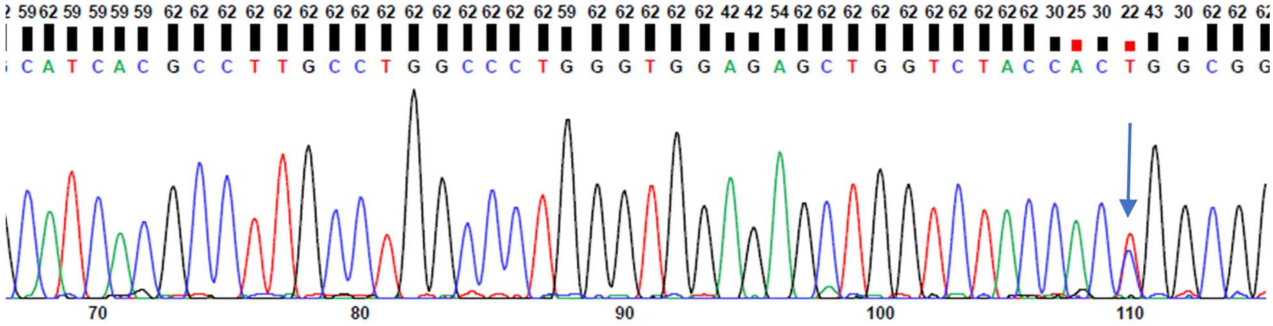

Supplementary tables

Supplement table S1. Supplement table S1.: Genes, included in our gene panel

Genes, used in the gene panel
APP','PSEN1','PSEN2','S100A9','SACS','CR1','BIN1','TREM2','CLU','CTNNA3','DNMBP','SORL1','BACE1','PICALM','GAB2','LRP6','ADAM10','ABCA7','CD33','TOMM40','MS4A4A','MS4A6E','TM2D3','CD2AP','EPHA1','CASS4','PLD3','INPP5D','DSG2','CDH12','CDH18','MEF2C','NME8','PTK2B','SLC24A4','RIN3','ZCWPW1','ACE','MTHFD1L','PINK1','PARK7','PARK9','ATP13A2','GBA','SNCA','PARK2','LRRK2','ACMSD','CD157','BST1','FBXO7','FGF20','GAK','GIGYF2','GPNMB','HIP1R','PTPRD','LAMP3','PLA2G6','STBD1','STK39','STX1B','SYT11','VPS35','TDP43','TARDBP','ATXN2','EWSR1','HNRNPA2B1','PFN1','SETX','TMEM106B','CCNF','PPT1','TBK1','DCTN1','NEK1','CHMP2B','SQSTM1','SIGMAR1','VCP','FUS','GRN','MAPT','TAF15','ALS2','UBQLN2','FIG4','OPTN','DAO','HNRNPA1','ANG','VAPB','SOD1','SPAST','CYP7B1','SPG11','CSF1R','NOTCH3','HTRA1','CTSA','PRNP','STX6','GAL3ST1','PDIA4','BMERB1','LPA','FGF20','TET1','ACO1','POSTN','ABCA13','ADAM18','ARAP2','ATP13A3','C1orf51','CIART','DDX1','FAM105A','FGF20','

FNDC1','GBP4','GPR63','HACL1','MYOZ2','PDLIM5','PKP1','POLR1C','SFRP4','SLAIN2','SLC6A5','SMC5','TWF1','VEPH1','WDR64','ZRANB2','ATXN1'

,'NOTCH4','GLA','COL4A1','COL4A2','FOXC1','FOXF2','TREX1','PMF','ZCCHC14','WDR123','ICAL1','GCH1','S2B3','ACOX1'

Supplement Table S2: Variants, found in the patient. The five variants of interest have been highlighted

Gene	HGVS.p	Zygosity	rs ID	p3_1000G	p3_1000G_EAS	gnomAD_exomes	gnomAD_exomes_EAS	SIFT_score	SIFT_pred
ABCA13	p.Arg555His	HET	rs2361519	0.198083	0.1508	0.145723	0.169234	1	T
ABCA13	p.Ile767Ser	HET	rs17712293	0.194489	0.2153	0.173606	0.224242	0.007	D
ABCA13	p.Phe1540Leu	HET	rs17712299	0.096446	0.1498	0.07085	0.179048	0	D
ABCA13	p.Ala2178Glu	HOM	rs1880736	0.812899	0.9881	0.723538	0.989164	1	T
ABCA13	p.Lys2306Thr	HET	rs78334925	0.097244	0.1508	0.075345	0.186615	0.11	T
ABCA13	p.Arg2674Trp	HOM	rs2222648	0.852236	0.9921	0.803456	0.991853	1	T
ABCA13	p.Thr4550Ala	HET	rs78734445	0.002596	0	0.004008	0	0.112;0.084	T
ABCA7	p.Arg1349Gln	HET	rs3745842	0.390575	0.3482	0.427897	0.335604	0.546;0.546;.	T;T;.
ABCA7	p.Arg1413Arg	HET	rs881768	0.442093	0.3552	.	.	.	.
ABCA7	p.Gly1527Ala	HET	rs3752246	0.825479	0.6478	0.840229	0.646508	0.877;0.877;.	T;T;.
ABCA7	p.Val1946Met	HET	.	.	.	.	.	0.031;0.031;.;.	D;D;.;.
ABCA7	p.Ala2045Ser	HET	rs4147934	0.605032	0.4325	0.71193	0.446435	0.962;0.962;.;.	T;T;.;.
ACOX1	p.Ile312Met	HET	rs1135640	0.550719	0.7877	0.653791	0.800441	0.256;0.201	T
ALS2	p.Val368Met	HOM	rs3219156	0.896565	1	0.912953	0.999826	0.191;0.172	T
ARAP2	p.Arg1523Gln	HOM	rs4833069	0.995208	1	0.992128	1	1.0;.	T;.
ARAP2	p.Pro89Leu	HET	rs35822395	0.005591	0	0.008379	0.000116	0.094	T
ATP13A2	p.Pro1122Leu	HET	rs15786	0.013179	0.001	0.04005	0	0	D
ATP13A2	p.Ala1072Thr	HET	rs3170740	0.33127	0.246	0.47141	0.295664	0.988;1.0	T
ATP13A2	p.Leu437Val	HET	rs149372969	0.000599	0	0.00106	0	0.219;0.588;0.236;.;0.544	T;T;T;.;T
ATXN1	p.Pro753Ser	HET	rs16885	0.122804	0.0069	0.195486	0.008407	0.015	D
ATXN1	p.Gln225dup	HOM	rs769091061	.	.	.	.	.	.
ATXN2	p.Pro425Leu	HET	.	.	.	.	.	0.001;.;0.001;.;0.002;0.001;0.001;0.003	D;.;D;.;D;D;D;D
ATXN2	p.Ser248Asn	HET	rs7969300	0.179513	0.5228	0.076308	0.564288	.;0.136;0.128	.;T;T
CD33	p.Arg69Gly	HOM	rs2455069	0.335064	0.1042	0.348885	0.080424	0.334;0.335	T
COL4A1	p.Gln1334His	HET	rs3742207	0.288339	0.2629	0.304165	0.251565	0.122	T
COL4A2	p.Arg517Lys	HOM	rs7990383	0.509185	0.2232	0.523827	0.230619	1	T
CR1	p.His1658Arg	HET	rs2274567	0.294329	0.3274	0.250551	0.291633	0.897;0.897;0.897;0.897;0.309	T

CR1	p.Thr2060Ser	HOM	rs4844609	0.995008	1	0.985426	1	0.804;0.804;0.804;0.804;1.0	T
CR1	p.Ile2065Val	HET	rs6691117	0.493411	0.3433	0.321622	0.3062	1	T
CR1	p.Pro2277Arg	HET	rs3811381	0.26278	0.3224	0.241761	0.288787	0.446;0.446;0.446;0.446;0.394	T
CR1	p.Thr2419Ala	HOM	rs2296160	0.828075	0.6885	0.819882	0.655775	0.987;0.987;0.987;0.987;1.0	T
CTSA	p.Leu37del	HOM	rs10582052,rs3080212,rs11468075	0.649361	0.5784	.	.	.	.
DSG2	p.Arg773Lys	HET	rs2278792	0.240016	0.4643	0.265563	0.466526	0.383	T
EPHA1	p.Met900Val	HOM	rs6967117	0.960264	1	0.940039	0.998493	1	T
EPHA1	p.Arg492Gln	HET	rs11768549	0.00619	0	0.012483	0	0.599	T
EPHA1	p.Val160Ala	HOM	rs4725617	0.94349	0.9921	0.93221	0.991984	0.246	T
FNDC1	p.Ser36Pro	HET	rs295332	0.550919	0.628	0.540541	0	0.981	T
FNDC1	p.Thr438Ala	HET	rs509648	0.508586	0.7232	0.324572	0.751479	1	T
FNDC1	p.Glu463Gln	HOM	rs420137	0.784545	0.621	0.866743	0.637705	1	T
FNDC1	p.Gln1003Glu	HOM	rs370434	0.785743	0.626	0.867677	0.641117	1	T
FNDC1	p.Asp1180Glu	HOM	rs420054	0.782348	0.626	0.850326	0.622257	1	T
FNDC1	p.Leu1261Pro	HOM	rs3003174	0.786142	0.626	0.86882	0.641139	0.345	T
FNDC1	p.Gln1280Arg	HOM	rs2501176	0.786142	0.626	0.869494	0.643025	1	T
FNDC1	p.Thr1479_Thr1484del	HOM	rs141435210	0.496406	0.4058	.	.	.	.
FNDC1	p.Thr1504Lys	HOM	rs369362288	0.784944	0.625	0.872142	0.644028	0.795	T
FOXC1	p.Gly380dup	HET	rs76840944	0.152157	0.2867	.	.	.	.
FOXF2	p.Gly306dup	HOM	rs147426137,rs58230522,rs111257067	0.392772	0.497	.	.	.	.
GBP4	p.Glu551Gly	HOM	rs561042	0.357428	0.3978	0.442432	0.399049	0.036	D
GBP4	p.Glu551Lys	HOM	rs561037	0.358826	0.3978	0.441993	0.398608	0.505	T
GBP4	p.Leu549Met	HOM	rs608339	0.359425	0.3988	0.43821	0.395317	1	T
GBP4	p.Glu546Lys	HOM	rs1142890	0.359026	0.3988	0.428369	0.38529	0.929	T
GBP4	p.Met545Ile	HOM	rs1142889	0.359026	0.3988	0.427021	0.384029	0.072	T
GBP4	p.Met545Leu	HOM	rs1142888	0.359026	0.3988	0.425834	0.382578	1	T
GBP4	p.Met542Ile	HOM	rs1142886	0.359625	0.3998	0.426034	0.381004	1	T
GBP4	p.Tyr541Asn	HOM	rs655260	0.359625	0.3998	0.430646	0.386015	1	T
GRN	p.Ala505Gly	HET	rs780159686	.	.	7.31E-05	0	0.257;.	T;.
LAMP3	p.Ile318Val	HET	rs482912	0.496406	0.4712	0.651055	0.506262	1	T
LRP6	p.Val1062Ile	HET	rs2302685	0.885583	0.9196	0.850649	0.9335	1	T
LRRK2	p.Arg50His	HOM	rs2256408	0.969249	1	0.993066	1	1	T
LRRK2	p.Met1646Thr	HET	rs35303786	0.004792	0	0.009376	5.81E-05	0.159	T
LRRK2	p.Met2397Thr	HET	rs3761863	0.551717	0.4603	0.618476	0.46794	0.466	T
MAPT	p.Tyr441His	HET	rs2258689	0.312899	0.628	0.281649	0.632031	0.978;0.933;0.978;.	T;T;T;.
MS4A6E	p.Ile6Val	HOM	rs2304935	0.258187	0.254	0.325262	0.270819	1	T
MS4A6E	p.Thr10Ala	HOM	rs2304934	0.258187	0.255	0.325243	0.27093	0.086	T
MS4A6E	p.Val47Phe	HOM	rs2304933	0.257788	0.255	0.325162	0.270275	0.044	D
NME8	p.Cys208Arg	HOM	rs10250905	0.743411	0.5466	0.731553	0.560475	0.046	D
NOTCH3	p.Ala2223Val	HOM	rs1044009	0.629393	0.5724	0.715381	0.584804	0.175	T

PARK2	p.Val380Leu	HET	rs1801582	0.172125	0.0853	0.164749	0.076357	0.377;0.416;0.607;0.335;0.335;.	T;T;T;T;T;.
PARK2	p.Ser167Asn	HET	rs1801474	0.117412	0.3393	0.069934	0.391814	0.219;0.229;0.155	T
PDLIM5	p.Ser14Phe	HET	rs2452600	0.224641	0.378	0.265912	0.374971	0.024;.	D;.
PDLIM5	p.Ala374Thr	HOM	rs966845	0.993411	1	0.993729	1	.;.;1.0;0.702;.;0.709	.;.;T;T;.;T
PDLIM5	p.Thr410Ala	HET	rs7690296	0.38758	0.3998	0.44145	0.399779	.;.;1.0;1.0;.;0.433	.;.;T;T;.;T
PDLIM5	p.Ser521Asn	HOM	rs13107595	0.979633	1	0.989681	1	.;.;0.74;0.571;.;0.91	.;.;T;T;.;T
PSEN1	p.Glu318Gly	HET	rs17125721	0.005591	0	0.014617	5.80E-05	0.135;0.132;0.135;0.004;0.224	T;T;T;D;T
PTK2B	p.His447His	HOM	rs7005936	0.370208	0.1954	.	.	.	.
PTPRD	p.Thr781Ala	HET	rs72694737	0.016973	0.001	0.022152	0.000759	1	T
RIN3	p.Thr425Met	HET	rs3742717	0.292532	0.4415	0.241143	0.467308	0.08;.	T;.
RIN3	p.Gly972del	HOM	rs71721558,rs68153141,rs71698059	.	.	.	.	.	.
SETX	p.Ile1386Val	HOM	rs543573	0.55611	0.3621	0.736559	0.303514	0.872	T
SETX	p.Gly1252Arg	HOM	rs1183768	0.55611	0.3621	0.736471	0.30334	0.133	T
SETX	p.Asp1192Glu	HOM	rs1185193	0.640575	0.3681	0.769512	0.311333	0.377	T
SFRP4	p.Arg340Lys	HET	rs1802074	0.252995	0.2619	0.197331	0.243691	0.859	T
SFRP4	p.Pro320Thr	HOM	rs1802073	0.542732	0.5635	0.435974	0.550848	0.171	T
SLC6A5	p.Phe124Ser	HOM	rs1443548	0.793131	0.75	0.771566	0.760212	0.44	T
SLC6A5	p.Ala162Gly	HOM	rs1443549	0.99361	1	0.998607	1	1	T
SMC5	p.Val306Ile	HOM	rs1180116	0.855032	0.8532	0.88404	0.855722	0.655	T
SORL1	p.Glu270Lys	HET	rs117260922	0.007788	0	0.015071	0	0.008	D
SORL1	p.Gln1074Glu	HOM	rs1699107	0.984824	1	0.996113	1	0.168;1.0	T
SORL1	p.Val1967Ile	HOM	rs1792120	0.979433	1	0.996169	1	1	T
SYT11	p.Gln48His	HOM	rs822522	0.954673	1	0.990874	0.999942	0.866	T
TET1	p.Asp162Gly	HET	rs10823229	0.258586	0.3919	0.330584	0.383148	0.019	D
TET1	p.Cys832Tyr	HET	rs74925160	0.024161	0	0.005267	0	0	D
TET1	p.Ala985Ser	HET	rs74412312	0.024161	0	0.00526	0	0.013	D
TET1	p.Ile1123Met	HET	rs3998860	0.693291	0.8502	0.777419	0.839626	0.157	T
TM2D3	p.Leu6Arg	HOM	rs2939587	0.993411	1	0.97935	1	0.311;0.318;0.466;0.404	T
TMEM106B	p.Thr185Ser	HET	rs3173615	0.595048	0.6508	0.489626	0.657474	0.214	T
TOMM40	p.Arg239Trp	HET	rs142412517	0.0002	0	0.0005	5.80E-05	0.011;0.011;.;0.011	D;D;.;D
VEPH1	p.Ser522Pro	HET	rs11918974	0.250599	0.3512	0.276265	0.392621	1.0;0.992;0.992	T
WDR64	p.Asn521Asn	HET	rs10802990	0.590855	0.5149	.	.	.	.
ZCCHC14	p.Val693Met	HOM	rs3748400	0.461462	0.3115	0.643568	0.307451	0	D
